# The ‘connectaholic’ behind the curtain: medical student use of computer devices in the clinical setting and the influence of patients

**DOI:** 10.1186/s12909-019-1811-8

**Published:** 2019-10-17

**Authors:** Eric Clarke, Jane Burns, Catherine Bruen, Martina Crehan, Erica Smyth, Teresa Pawlikowska

**Affiliations:** RCSI Health Professions Education Centre, Royal College of Surgeons in Ireland, 123 St. Stephen’s Green, Dublin 2 Dublin, Ireland

**Keywords:** Technology enhanced learning, Medical education, Medical students, Clinical placement, Portable devices

## Abstract

**Background:**

The use of mobile devices such as tablets and laptops by students to support their learning is now ubiquitous. The clinical setting is an environment, which lends itself to the use of mobile devices as students are exposed to novel clinical scenarios that may require rapid location of information to address knowledge gaps. It is unknown what preferences students have for these devices and how they are used in the clinical environment.

**Methods:**

In this study we explored medical students’ choices and their use of different devices in their first year of clinical attachments. We sought to evaluate learners’ experiences with these devices using a mixed methods approach. All students newly entered into the clinical years were given the option of a MacBook Air or iPad. We surveyed these students using an online survey tool followed by individual semi-structured interviews to explore survey findings in more depth.

**Results:**

Students owned a multitude of devices however their preferences were for the 11 in. MacBook Air Laptop over the iPad mini. Students made constant use of online information to support their clinical learning, however three major themes emerged from the interview data: connection and devices (diverse personal ownership of technology by students and how this is applied to source educational materials), influence and interaction with patients (use of any device in a clinical setting) and influence and interaction with staff. In general students preferred to use their device in the absence of patients however context had a significant influence.

**Conclusions:**

These mobile devices were useful in the clinical setting by allowing access to online educational material. However, the presence of patients, and the behaviour of senior teaching staff significantly influenced their utilisation by students. Understanding the preferences of students for devices and how they use their preferred devices can help inform educational policy and maximise the learning from online educational content.

## Background

Medical education has been transformed over the last decade with the introduction of student centered approaches to learning [1–3]. As part of this student centered approach, technology enhanced learning (TEL) has made digital devices and digital content essential requirements for the health professions student. The importance of technology in medical education was highlighted in the *NMC Horizon Report 2017* which suggests that online, mobile, and blended learning are inevitable, and suggests that embracing digital approaches to medical education is essential for individual medical school survival [4].

Whereas static deskbound internet connectivity was once commonplace in education, mobile devices and portable computers with continuous data connections now allow students to access information while on the move, which for medical students also encompasses clinical settings [5, 6].

While the potential for computers and TEL to influence fundamental changes to medical education are recognised, these innovations are often initially realised as general consumer products. By their nature, these products quickly appear in teaching settings and it may not be apparent whether the pedagogical imperatives of medical education are served by such developments. As a consequence, understanding of how best to use technological innovations and associated information sources to support heath care providers and patients continues to stimulate considerable debate [7].

Use and ownership of mobile devices such as smartphones, tablets and laptops amongst medical students has been reported to be relatively high and unsurprisingly, data suggests that this trend is increasing [8–12]. A selection of medical schools have embraced this concept of enhancing student learning with digital content and support this by providing students with portable devices such as tablets and laptops .

In the absence of an industry definition of a portable device, for the purposes of this study we defined a portable device as a device which is wireless, has internet connectivity and can be carried. Portable devices can provide students with a deluge of medical and educational resources almost instantly in a variety of contexts. Previous work has demonstrated that students find devices such as tablets are useful for their education [13, 14] and the use of such devices have been reported to improve exam performance [15, 16]. Furthermore, the use of portable devices has not only been shown to have a positive influence on students but it also has been shown to enhance patient encounters [13]. However, these findings are not universal as the evidence regarding the effectiveness of digital devices in medical education is contradictory. Several studies have reported that actual utilisation of devices for learning is low [17] and they have been suggested to cause disruption in teaching sessions [18, 19]. Moreover, laptops and mobile phones have both been reported by undergraduate medical students to disturb their concentration and ability to learn [20].

The clinical setting is a learning environment where students hone their clinical skills; interact with patients and senior staff, while consolidating and applying knowledge learnt in the classroom. Digital devices lend themselves to learning in this environment due to the dynamic and busy nature of the healthcare clinical context. Mobile phones and tablets are used by medical students to access resources such as drug information and clinical scoring systems, however it is unknown what students preferences are towards devices that are provisioned by medical schools such as laptops and tablets [21, 22].

In order for this digital movement in medical education to continue and evolve, and the use of digital education content to be used in the most effective way, we must gain a deeper understanding of the perspectives of medical students on their digital device preferences and their uses. In this study we explored clinical students’ choices and their use of different devices in their first year of clinical attachments. We sought to evaluate learners’ experience with these instruments using a mixed methods approach.

## Methods

### Participants

Incoming medical students at our institution are routinely provided with a PC laptop (Hewlett Packard) on commencement of their medical studies, and this is renewed on entry to their clinical years. In 2013 an institutional decision was made to offer students the choice of an 11-in. Mac Book Air or iPad with mobile broadband internet on entry to their clinical years (4th year students). Whilst the Mac Book Air was a smaller more portable laptop, it was felt that the iPad could be more amenable for immediate portability and access to information on clinical placements. Students were free to choose their preferred device and use them as they wanted whilst attending their initial clinical year. All students received this independently of any other devices they owned.

### Data collection

We surveyed all students newly entered into the clinical years using an online survey tool (Survey monkey) and two subsequent reminders were sent at intervals. The survey was based on a review of the relevant literature and previous institutional surveys. It explored students’ experience and decisions regarding device choice and use.

We used purposeful sampling - inviting a maximally variant sample (heterogeneity selected on the basis of device and self-reported use) of these students to individual semi-structured interviews (19 interviews) to explore the survey findings in more depth. (Staff carrying out interviews were not involved in clinical teaching). The students who were interviewed had comparable demographic data to the survey group. Interviews were conducted at the time and place of the student’s choosing and contemptoraneous field notes were made by interviewers. The interviewers, who were all part of the health professions education centre team (CB, JB and EC) underwent specific training by an educationalist, also part of this team, with extensive qualitative research experience (MC).

### Data analysis

Analysis of initial interviews indicated that use of devices in clinical settings by students was influenced by the presence of patients. Based on these findings, subsequent interviews were modified to explore this aspect in more detail. Students who did not a use device with patients were asked to explore their decision and to provide further insight into why the device was not used.

The survey data was analysed with SPSS version 22.0 ER. The semi-structured interviews were transcribed verbatim and analysed by inductive thematic analysis using the constant comparison method [23]. Interviews were initially double coded and memos were generated (JB, EC, TP). Interviewers discussed the analysis and agreed the coding and preliminary themes. Through an iterative process of coding and discussion, consensus was reached regarding the major themes.. Throughout this process, field notes and memos were reviewed and used to inform this analysis. Interviews continued until no new information emerged and saturation was reached. Findings were then subjected to respondent validation.

## Results

### Study demographics

Of the cohort of 318 clinical medical students, 279 (87.7%) students opted for a Macbook Air laptop and a total of 39 (12.3%) opted for an iPad. 93 (29.2%) students chose to answer the online survey, which was circulated in the second week of term. There was a 100% completion rate of all items on the survey. Demographics of study participants are described in Table 1; they were representative of the class as a whole.

**Table 1:** Demographics of survey group

Age (yrs,mean ± SD)	22.9 ± 4.5
**Gender**	
Female	41 (44.1%)
Male	52 (55.9%)
**Region**	
European	40 (43.0%)
North American	19 (20.4%)
Middle Eastern	16 (17.2%)
Far Eastern/Asian	12 (12.9%)
Other	6 (6/5%)

Demographics of the individuals who completed the survey. Subheadings are in bold. European - France, Germany, Irish, Norway and UK. Far east/Asia - Hong Kong, India, Korea, Malaysia, Pakistan, Philippines and Singapore. Middle East - Bahrain, Egypt, Iraq, Jordan, Kuwait, Oman, Saudi Arabia and U.A.E. North America - Canada, U.S.A. Other - Australia, Mauritius, Nigeria, South Africa, Trinidad. *N* = 93.

### Student device ownership and usage

The survey of ownership showed that all students had a smartphone but only 5% had a desktop computer (Table 2). However, in addition to the laptop or iPad issued by the medical school, students also possessed an array of personal electronic Internet enabled devices, including a second laptop (52%) or a tablet computer (44%) (Table 2). Almost all students reported daily use of technology for educational purposes (94.6%) with a small remainder of students (5.4%) using devices on a weekly basis. Daily use of technology reported for other purposes such as entertainment (83.9%), general web browsing (91.4%) and communication/social media (93.5%) was also high and reflects an almost constant connection to the Internet and online services.

**Table 2:** Ownership of electronic devices by students

Device	No. Of students	%
Smartphone (with Internet connection)	93	100%
Macbook Air	76	82%
Second laptop^a^	48	52%
Desktop computer	5	5%
iPad^a^	41	44%
Kindle/Nook	12	13%
Netbook	1	1%

The number and percentage of electronic devices owned by senior cycle medical students.

^a^personal purchase outside of College scheme

### Perceived helpfulness of iPad in clinical settings with or without patients present

50% of students using iPads noted that they never used the device in a clinical setting where a patient was present (Table 3). Of those students who did use an iPad with patients only 14.7% of students reported that the device was helpful. This contrasts with the majority (64.7%) who stated that iPads were helpful when patients were not present. IPads were used principally to retrieve theoretical clinical information to assist students. Data collected during qualitative interviews indicated that students were also using personally owned Internet enabled Smartphones to look up such clinical information.

**Table 3:** Perceived helpfulness of iPad in clinical settings with or without patients present

	Very helpful		Slightly helpful		Neutral		Slightly unhelpful		Very unhelpful		Did not use	
	N	%	N	%	N	%	N	%	N	%	N	%
Without patients present	13	38.2%	9	26.5%	5	14.7%	0	0.0%	1	2.9%	6	17.7%
With patients present	3	8.8%	2	5.9%	10	29.4%	1	2.9%	1	2.9%	7	0%

The number and percentage of students who perceived helpfulness of iPad in the clinical environment as very helpful, slightly helpful, neutral, slightly unhelpful, or did not use in the presence or absence of patients. *N* = 34.

### Student perspectives on mobile devices

The interviews time ranged from 14 to 54 min with an average time of 30 min. Interviews were conducted and analysed under the view that student perspectives are influenced by their experiences and this reality is complex and context-dependent. Three major categories were identified in the qualitative data:

1. Connection and devices (Diverse personal ownership of technology by students and how this is applied to source educational materials)
2. Influence and interaction with patients (Use of any device in a clinical setting)
3. Influence and interaction with staff

Within the three categories, two major themes were present concerning the use of devices and students perceptions of the appropriateness of their use in the presence of patients.

Themes

1. Devices are ideal for bed-side care
  Both the laptop and the iPad were considered devices that could be used at the bedside and in the clinical space (SN_7 and SN_1) (Table 4). The students felt that these devices were integral to their day-to-day activity and organisation (SN 3),” So I’m very dependent on my iPad. It helps me organize pretty much everything” (SN 3), and were essential for looking up educational sources and connecting to the internet; this brought a level of comfort to some subjects “It’s a horrible thing to be on the wards and not know what’s going on and not know what you’re dealing with. So it’s very comforting to have it, have the phone there and be able to go on straight away and look at things” (SN 15) (Table 4).
2. Appropriate and inappropriate use of devices
  The appropriate use of devices was a subject that was highlighted by many students (SN 16, SN 1, SN 11 and SN 15). Students perceptions of appropriate use of devices was conflicting, as some students thought the use of a device with patients present created a barrier “So I tend not to take it out, because I just think it kind of creates a barrier between me and the patient” (SN 15), while others found it very appropriate with certain patient groups “kids just love tablets” (SN 1) (Table 4)..
  Furthermore, staff also influenced the use of devices in the clinical setting. For the majority of students, staff were perceived to enforce implicit rules about using devices in front of patients “the registrar…..told me...just don’t take out your phones, if you need anything just ask me……that is kind of the rules and stuff.” (SN 14). However, in certain circumstances, the devices were seen to be helpful and aid in patient communication “..after the procedure was done, the surgeon then used the application and the diagrams of the (iPad) app to explain which muscles exactly he was cutting and which ligaments he was tightening, which approach he took, etcetera. So it was actually very useful.” SN 10 (Table 4).

**Table 4:** Students’ use of Internet enabled devices in clinical settings

Connection and devices	
SN_07	The laptop made my life… easier. I can take my laptop anywhere with me.
SN_03	So I’m very dependent on my iPad. It helps me organize pretty much everything.
SN_1	I found it really invaluable. I mean… when you’re on rotation, every once in a while you’ll get an hour or two when your team is not really up to anything. And besides, I guess carrying a textbook around with you all the time, there’s really no better way than just having the iPad..
SN_15	It’s a horrible thing to be on the wards and not know what’s going on and not know what you’re dealing with. So it’s very comforting to have it, have the phone there and be able to go on straight away and look at things.
SN_17	… I just get connected… maybe I get the urge… I’m a connectaholic.
Influence and interaction with patients	
SN_16	I don’t feel like it’s appropriate to use it in front of the patient, they might think that I’m just like playing with my iPad instead of doing something so.
SN_1	I feel like I might be able to use it maybe a little bit more now that I’m in paediatrics. I’ve been meaning to try it, sort of just like to entertain the kids, get them on your side, that type of thing. Just because kids love tablets.
SN_11	I think it would be strange to type in front of the patient firstly, and I think it interferes with the rapport.
SN_15	I find that the second that the phone comes out in the clinical setting, that the patient just sort of…, a barrier goes up. I think they fear that we’re not paying attention to them if the phone is there. So I tend not to take it out, because I just think it kind of creates a barrier between me and the patient, and I don’t like that.
SN_17	(the clinical teacher) was busy examining and talking with the patient, so I just went behind the curtain and just Googled it quick.
Interactions with staff	
SN_12	... like the doctors will tell you…..It’s unprofessional to use your phone. Older patients especially won’t appreciate it.
SN_14	the registrar…..told me...just don’t take out your phones, if you need anything just ask me……that is kind of the rules and stuff.
SN_09	I would say it would be more comfortable using it in a GP setting where you have your own desk talking to a patient or like an out-patient clinic probably, more than in the ward .
SN_06	….you know just being on the delivery ward we’re with one patient for quite a long time and … and even if they’re asleep they are saying (staff) use of mobile phones is a big ‘no no’ is actually the wording they use ... you know we have a kind of list of rules and regulations and that’s one of them.
SN_10	..after the procedure was done, the surgeon then used the application and the diagrams of the (iPad) app to explain which muscles exactly he was cutting and which ligaments he was tightening, which approach he took, etcetera. So it was actually very useful.

Representative quotes describing student perspectives on the use of laptops and iPads in the clinical setting. Themes are categorised into connection and devices, influence and interaction with patients and interactions with staff. SN = Study Number.

## Discussion

This study provides insight into how medical students use technology in clinical settings. Student use of technology in these settings revealed ownership of an eclectic array of devices applied in a multitude of ways for learning. While devices are commonplace and heavily used by students, students are reticent in using them due to personal convictions and mixed messages regarding correct protocol from staff. This study portrays students as being online, mobile and fluently exploiting data and services from a wide variety of sources in both their academic and personal lives. While the presence of technology in a medical student’s life may appear to be ubiquitous, our results also highlight differences when technology is used in clinical settings with or without patients.

While students bring a variety of devices into the clinical setting, their actual use is linked to personal feelings based on the student’s perceptions of how patients will view the use of the device in terms of acceptance and professionalism. Our data indicate that students are purposively selective as to how and when technology is used in clinical settings. This selectivity is mediated by students’ personal convictions on how the use of devices may affect patients’ perceptions of them and their consultation. These convictions may be responsible for the findings by Chase et al., that students were likely to use their devices in “down-time” rather than as part of their clinical session [14].

All students in our study were more or less continuously connected and engaging with clinical information online to support their studies. One study participant ascribed the moniker of “connectaholic”, our data shows that this is influenced by current students’ personal habits, all of which have implications for medical education, particularly so in clinical settings.

The thematic analysis revealed that while students are cautious about using devices in front of patients, they highly valued their educational and organizational utility. The analysis also revealed that student’s perception of what was appropriate and inappropriate behavior concerning device use, relied heavily on context and the individuals in their environment.

Our findings mirror those of [14, 24, 25] in that students possess an array of personally purchased devices and actively use them to reference information in a variety of ways. Results are consistent with other studies which found near complete ownership of smartphones [26]. This is a phenomenon that many clinical academics will readily recognise. In our own medical school, the longstanding compulsory laptop/iPad scheme (since 1993) also promotes the use of technology as a matter of policy (namely that all students are issued with a device).

While the presence of technology in a medical student’s life appears to be ubiquitous, our results also highlight differences when technology is used in clinical settings with or without patients being present. This difference in use is influenced by factors such as personal and non-academic experiences, and is rooted in the subsequent formation of personal convictions that using electronic devices with a patient is impolite or may be viewed as unprofessional. These results support and contribute to the growing body of evidence that patients or other members of clinical staff significantly influence the use of mobile devices by medical students [27]; This highlights the need for a clarification and consensus on the use of devices at an organizational level, for example in the form of explicit guidelines. Moreover, as student experiences with the devices are largely positive and *appear* to promote learning, defining how these devices are used in the clinical environment would enhance student learning.

Studies evaluating the use of the iPad as an educational tool in clinical settings have reported mixed results [14, 28], however the use and presence of these devices cannot be ignored. As medicine becomes more complex, learners are required to look up information at the point of patient care [29]. Associated with the need for access to information, students in higher education are increasingly armed with an eclectic array of electronic devices such as laptops, tablets, or smartphones [30].

It may not be possible to consider any group of students as a single homogenous entity with distinct preferences for use of technology in their learning [25], however there is a need to recognise that as medicine becomes more complex, learners are increasingly required to reference relevant information at the point of patient care [29].

The arrival of technological devices into the classroom has also blurred the boundaries of propriety [31] and there is now a further and progressing dimension to this debate, which must be considered as we allow seamless incorporation of technology into our daily professional lives. The use of mobile technology may add to the potential asymmetry in clinical settings and students must also learn to identify these issues and how to deal with them. Student’s use of technology has an influence in their use in clinical settings as students to “go behind the curtain” to reference information.

Guidance to students from teaching staff on the appropriate use of devices is clinical settings can be variable, ranging from total prohibition to direct and open use to support teaching. This was evidenced by the conflicting situations where staff enforced “no device” rules while also using a device to communicate with patients. This variability leads to confusion and loss of potential, which should be addressed by institutional guidelines and clear policy [32]. Findings from the current study align with the recent evidence based BEME Guide No. 52 highlighting the need for explicit policies to be put in place to tackle any uncertainty amongst students regarding device usage in the clinical setting [27].

The issues within this space are complex and there are some similarities to be drawn here between clinical and social settings. The problem arises from the fact that the same device that is such a powerful clinical tool is also connected to social media, email, and other non-medical outlets, posing the potential for inappropriate use in the workplace [33, 34]. Mobile technology is disruptive by its very nature and use in some social settings may be considered inappropriate or have a negative impact. Indeed, devices have been reported to cause disruption and be distracting to learners [18–20] and cause distractions in educational sessions like clinical rotations [11, 35, 36].Answering calls, sending and receiving text messages and updating social media can happen in any location.

### Limitations

The survey response rate in this study (29.2%)similar to many electronic surveys, was low and the possibility of responder bias needs to be taken into consideration*.* During data collection the study was contextualised as being focused on the devices delivered by the institution. The dynamics of the interview were such that students felt enabled to mention associated devices e.g. their mobile phone. One of the limitations of this study is that we were unable to fully differentiate between the use of the tablet and laptop, which the students were allocated, and those that the students themselves owned. It is possible that the perceived benefits of these devices by the students, could be due to personal devices and they may overstate the importance of institutionally provided devices. Furthermore, the financial value of the devices cannot be ruled out as an influence in the selection of student devices.

## Conclusion

We investigated a cohort of medical students given the choice of Macbook air laptops or iPads to support their learning on entry to their clinical years. Smartphone use for Internet connection was ubiquitous and students made constant use of online information to support their clinical learning. However, actual patterns of students’ use of various devices and online information are not uniform and are deeply influenced by the presence of patients, which students usually find inhibitory and this is coupled with a sometimes confusing variety of approaches modelled by clinical teaching staff.

## Data Availability

The datasets used and/or analysed during the current study are available from the corresponding author on reasonable request.

## Abbreviation

TEL: Technology Enhanced Learning
